# Empirical impact evaluation of the WHO Global Code of Practice on the International Recruitment of Health Personnel in Australia, Canada, UK and USA

**DOI:** 10.1186/1744-8603-9-60

**Published:** 2013-11-14

**Authors:** Jennifer S Edge, Steven J Hoffman

**Affiliations:** 1 Harvard Global Health Institute, Harvard University, Cambridge, Massachusetts, USA; 2 Department of Clinical Epidemiology & Biostatistics and McMaster Health Forum, McMaster University, Hamilton, Ontario, Canada; 3 Department of Global Health & Population, Harvard School of Public Health, Harvard University, Cambridge, Massachusetts, USA

**Keywords:** Health worker recruitment, Migration, Health systems, International law, Impact evaluation, World Health Organization

## Abstract

**Background:**

The active recruitment of health workers from developing countries to developed countries has become a major threat to global health. In an effort to manage this migration, the 63^rd^ World Health Assembly adopted the World Health Organization (WHO) Global Code of Practice on the International Recruitment of Health Personnel in May 2010. While the Code has been lauded as the first globally-applicable regulatory framework for health worker recruitment, its impact has yet to be evaluated. We offer the first empirical evaluation of the Code’s impact on national and sub-national actors in Australia, Canada, United Kingdom and United States of America, which are the English-speaking developed countries with the greatest number of migrant health workers.

**Methods:**

42 key informants from across government, civil society and private sectors were surveyed to measure their awareness of the Code, knowledge of specific changes resulting from it, overall opinion on the effectiveness of non-binding codes, and suggestions to improve this Code’s implementation.

**Results:**

60% of respondents believed their colleagues were not aware of the Code, and 93% reported that no specific changes had been observed in their work as a result of the Code. 86% reported that the Code has not had any meaningful impact on policies, practices or regulations in their countries.

**Conclusions:**

This suggests a gap between awareness of the Code among stakeholders at global forums and the awareness and behaviour of national and sub-national actors. Advocacy and technical guidance for implementing the Code are needed to improve its impact on national decision-makers.

## Introduction

Developing countries face a shortage of 4.3 million health workers that has long been exacerbated by the migration of their domestically-trained health workers to developed countries [1]. The effect of “push” factors like poor working conditions in source countries, combined with the attractive “pull” factors like higher wages in destination countries, encourages the migration of health workers from the areas in which they trained to countries with greater opportunities (see Table 1) [1-15]. This migration no doubt poses a serious ethical, political and legal dilemma for developing countries between their need to retain the health workers they train and their obligation to respect the international human right to freedom of movement and health workers’ right to choose where they want to live and work [16-21]. Individual health workers may also face their own dilemma between pursuing the best living circumstances for themselves and their families and their moral obligation to provide health services to those who most desperately need them.

**Table 1:** Summary of “Push” and “Pull” factors on the migration of health workers

Push factors encouraging emigration from source countries	• Poor remuneration [1-15]
	• Concerns for personal safety [1,2,4,5,12,13]
	• Few career prospects and opportunities for promotion [1,4-8,12-15]
	• Poor working conditions and heavy workload [1,4-10,12-15]
	• Poor living conditions [1,2,5,6,8,12,14]
Pull factors encouraging immigration to destination countries	• Better remuneration [1-3,5-7,9-15]
	• Safer environment [1,2,4,5,12]
	• Professional development and career advancement opportunities [1,4-8,12,14,15]
	• Improved working conditions and facilities [1,4-10,12,14,15]
	• Higher standards of living [2,5,6,8,12,14]

However, the active recruitment of health workers by developed countries encourages and deepens this migratory pattern by influencing health workers’ decisions to emigrate from their source countries, resulting in unnecessarily severe shortages of health workers in certain areas and leaving millions of people without access to health services [1,20,21]. According to the World Health Organization (WHO), this active recruitment and the resulting migration of health workers has become one of the greatest threats to global health in the 21^st^ century [1]. Indeed, many developed countries, such as Australia, Canada, United Kingdom (UK) and United States of America (USA), have chronically deficient health workforces and have only been able to sustain their relatively high health worker-to-population ratios by actively recruiting doctors, nurses and other health workers from developing countries, including those in Sub-Saharan Africa which is the region with the world’s greatest shortage [22-29]. The inequitable distribution of health workers is highly apparent. The Americas, for example, bear only 10% of the global disease burden, but have 42% of the world’s health workers. Sub-Saharan Africa, in contrast, carries 25% of the global disease burden but has just 3% of the world’s health workers. Over 50% of this region’s countries do not meet WHO’s acceptable physician-to-population ratio of 1 per 5000 [1]. Given these disparities, investing in domestic health worker training and retention, and discouraging the emigration of health workers, has become vital to strengthening health systems in developing countries [1,30].

The need to address this global shortage and inequitable distribution of health workers was prominently identified at least as far back as the Declaration of Alma-Ata in 1978, which emphasized the importance of health workers to functioning health systems [31]. Recent intergovernmental declarations have also called for greater regulation to ensure that all types of health workers are recruited “ethically” from developing countries. These include the:

1) World Organization of Family Doctors’ Melbourne Manifesto: Code of Practice for the International Recruitment of Health Care Professionals (2002); [32]

2) Commonwealth Code of Practice for the International Recruitment of Health Workers (2003); [33]

3) UK Department of Health’s Code of Practice for the International Recruitment of Healthcare Professionals (2004); [34]

4) World Federation of Public Health Associations’ Code of Ethics Pertaining to Health Worker Recruitment from Developing Countries (2005); [35] and

5) Pacific Code of Practice for Recruitment of Health Workers (2007) [36].

Professional associations and at least one government have adopted similar profession-specific guidelines, including the Australian Nursing Federation, [37] International Council of Nurses [38], World Medical Association, [39] and Ireland's Department of Health and Children [40]. Yet despite these resolutions, the active recruitment of health workers continued [23-25]. Urgent calls from the global community were then issued to regulate the international recruitment of health workers with new global guidelines that would be applicable to all countries and types of health workers [1,20-23,25,29,41-44].

Building on the efforts of previous declarations, the 63^rd^ World Health Assembly adopted the WHO Global Code of Practice on the International Recruitment of Health Personnel in May 2010 which became the first globally-applicable regulatory framework for international health workforce recruitment. The Code states that all Member States should aim to create a sustainable health workforce though planning, education and training, and retention such that their need to recruit migrant health workers is reduced. Bilateral arrangements should promote the provision of technical assistance, support health worker retention, ensure that training in source countries that is congruent with the country’s disease profile, encourage the twinning of health facilities, develop adequate regulatory frameworks and support return migration and technology and skills transfers (see Table 2) [45].

**Table 2:** Key elements of the WHO Global Code of Practice on the International Recruitment of Health Personnel

**Goal**	**Specific elements**
Establish ethical framework	Establishes ethical framework for international health worker recruitment based on voluntary principles
Balance rights	Balances the rights, obligations and expectations of source and destination countries and health workers
Strengthen health systems	High-income countries should support health systems strengthening through voluntary financial means, and provide technical assistance, training, technological and skill transfer and promote circular migration to create a net positive effect on low-income source countries
Support domestic development	Prioritizes the development of domestic health personnel and managing the mal-distribution of health workers between rural and urban areas
Facilitate information exchange	Calls for the creation of bilateral agreements, a national database of laws and regulations, designation of a national authority responsible for exchanging information with the WHO Secretariat and research partnerships at national, sub-national, and international levels
Develop regulatory framework	Supports capacity building for health information systems, continuous monitoring and evaluation of the health labour market and the development of a regulatory framework for health worker retention
Encourage compliance	Urges that the Code’s contents be publicized among all stakeholders involved in health worker migration and that governments only interact with recruitment agencies that operate in compliance with the Code
Enhance training	Recommends that training in source countries match the disease profile of such countries, encourages the twinning of health facilities, and demands that access to specialized training and technology be made a priority

The Code’s inclusion of all countries, sectors and types of health workers makes it distinct from previous declarations by filling the perceived gaps among the patchwork of previous country-, region- and profession-specific instruments (see Table 3) [43,46]. It also serves as the first universally-accepted set of ethical standards for national and sub-national actors involved in health workforce recruitment [47]. While technically the Code is not legally binding and has no enforcement mechanism, it may still constrain future decision-making through political pressure and by setting norms that are socially desirable to follow. The Code could also become legally binding in the future by incorporation into global conventions or international trade treaties, or it could become part of customary international law through the combination of state practice and *opinio juris* (i.e., the sense of obligation that the law requires states to act in this way). The recent Bangkok Outcome Statement of the Second Global Forum for Human Resources for Health (2011) demonstrates continued support for the Code and stakeholders’ belief in its ongoing relevance [48].

**Table 3:** Comparing the various current international codes on health workforce recruitment

**CODE**	**Stated objectives**	**Scope**	**Implementation mechanism**	**Considerations for developing countries**	**Distinguishing features**
**WHO Global Code of Practice on the International Recruitment of Health Personnel (May 2010)**	Establish and promote voluntary principles; Serve as a reference to improve legal framework; Provide guidance in the formulation and implementation of bilateral agreements; Facilitate and promote international discussion and cooperation	Global	Bilateral agreements among states and other supplementary international legal instruments	Destination countries should respect the overriding legal obligation of health personnel to fulfill their working obligations in home countries and seek not to recruit them	Establishment of national health authority to provide updates on Code implementation and exchange information on health workforce migration to the WHO Secretariat
				Destination countries should provide financial and technical support to developing source countries	Global scope: considers rights and obligations of both source and destination countries and migrant health personnel
**WFPHA Code of Ethics Pertaining to Health Worker Recruitment from Developing Countries (May 2005)**	Judiciously manage the employment of health professionals from abroad	International—applies to all member states of the WFPHA	Mandating WFPHA governments work only with employers that comply with the Code	Low-income countries receive something in compensation for sending health professionals (e.g. health worker exchange programs, government remuneration, continuing education for workers)	Builds upon UK DoH Code of Practice by restricting recruitment from developing countries that only have bilateral agreements with WFPHA
					Proposes definition for “active recruitment”
**UK Department of Health Code of Practice for the International Recruitment of Healthcare Professionals (Dec 2004)**	Offer principles and best practice benchmarks to be met in order to supply and manage international health professionals in an ethical manner.	Regional – applies to employers of the UK’s National Health System	Mandating NHS to work only with recruitment agencies that comply with the Code	Aims to prevent the active recruitment of healthcare workers from developing countries unless a government-to-government agreement to support recruitment exists	First national code of practice for international recruitment
	Provide targeted recruitment guidelines, education and language proficiency requirements, and employment laws related to international recruitment in order to establish ethical practice (DOH, 2004).			Manages migration with respect to active recruitment, but does not advocate for the retention or training of health workers in either the source or destination country	Best practice benchmarks to gauge adherence to core principles
					Online registry of commercial recruitment agencies complying with the code of practice
					Non-compliance by recruitment agencies can lead to grievances, investigations and loss of business with NHS.
**Commonwealth Code of Practice for the International Recruitment of Health Workers (May 2003)**	To provide Commonwealth governments with a framework for the ethical international recruitment of health workers to take place, taking into account the impact of such recruitment on source countries	International – applies to all governments of the Commonwealth nations	Promote dialogue among developed and developing countries to resolve this challenge	Acknowledges that recruitment diminishes the source country’s human resources and negatively impacts health systems.	Proposes its scope go beyond Commonwealth nations and be taken as a proposed global code of practice on this issue
			Follow-up with bilateral and other contractual agreements, e.g. bonding health workers	Bilateral agreements should regulate the recruitment process and be accompanied by mechanisms to detect non-compliance. (Labonte, Packer et al, 2007).	

While the Code has been lauded as an important development in the regulation of international health worker recruitment [45] its impact on national and sub-national actors’ behaviour has yet to be evaluated. This question is particularly important due to the proliferation of non-binding declarations issued by the global health community and used in global governance more broadly [49,50]. Furthermore, the immense financial and opportunity cost of developing these global codes and their implementing devices warrants an evaluation of their ability to effectively impact national decision-making. This study provides the first empirical evidence for whether the Code has influenced the behaviour and decisions of national and sub-national actors across all sectors involved in international health worker recruitment.

### Study design and methods

This study involved a survey of key informants in Australia, Canada, UK and USA from across government, civil society and private sectors to measure awareness for and perceived impact of the Code and its implementation. These countries are the four English-speaking developed countries with the greatest number of migrant physicians and nurses [12,28]. Government, civil society and private sectors were surveyed to reflect the inter-sectoral, multi-stakeholder implementation approach described in the Code (Article 5.6). We employed a mixed-methods approach to the content analysis, drawing on the mostly-qualitative survey responses to identify key themes and extract quantitative summary statistics.

### Questionnaire design

The questionnaire consisted of nine targeted questions probing key informants’ awareness of the Code, changes resulting from it, ways to improve its implementation, and key informants’ overall opinion on the effectiveness of non-binding codes in general (see Web Additional file 1 to view the questionnaire). Eight questions were open-ended and one question asked participants to rate their level of agreement with a statement about the impact of the Code on a 7-point Likert scale ranging from “Strongly Disagree” (1) to “Strongly Agree” (7).^a^ Questions were informed by a comprehensive literature review and developed based on how the Code would be translated into policy and practice. The questionnaire was refined in consultation with WHO staff who specialize in health workforce migration.

### Data collection

A sampling frame of 334 individuals that were directly involved in regulating, setting policies about, and/or practicing the active recruitment of health workers from developing countries was assembled using purposive internet searches, snowball sampling, and the invitee list for the Second Global Forum on Human Resources for Health. Questionnaires were distributed by email and followed by two reminders throughout January-March 2011, which was 8–10 months after the Code’s adoption by the World Health Assembly. This timeframe allowed researchers to analyze the short-term impact of the Code on decision-making by gauging individuals’ awareness of the Code within 12 months of its adoption. The goal was to receive responses from individuals representing each of government, civil society and private sectors in the four countries.

### Data analysis

Qualitative survey responses were coded through an iterative process and analyzed using grounded theory methodology for common themes and trends across sectors and countries. Quantitative descriptive statistics were extracted from the qualitative data through further content analysis and representative quotations from the key informants were identified.

### Ethics approval

This study was approved by the McMaster University Faculty of Health Sciences/amilton Health Sciences Research Ethics Board in Hamilton, Ontario, Canada.

## Results

Responses were received from 42 key informants with nearly every sector represented in each of the four countries (see Table 4). Government respondents were from national ministries of health and regulatory bodies responsible for licensing health workers. Private sector respondents were from consultancies and health worker recruitment agencies. Civil society respondents were from policy institutions, academia, and national trade unions. Job titles of the key informants included Human Resource Manager, Associate Dean, Chief Medical Officer, President, and Chief Executive Officers, among others.

**Table 4:** Number of survey respondents by country and sector

	**Government**	**Civil society**	**Private sector**	***Total***
**Australia**	2	6	2	10
**Canada**	1	9	0	10
**UK**	0	5	1	6
**USA**	1	10	5	16
*Total*	4	30	8	42

### Awareness for the code

Sixty percent of respondents believed their colleagues were not aware of the Code (n = 25). As articulated by one American respondent from the private sector:

“I am not familiar with WHO’s Global Code of Practice on International Recruitment of Health Personnel. I also believe that many organizations like ours are not [aware of it], as I have never heard this code mentioned by any of them” (US.PS.03).

Of the 17 respondents who reported awareness for the Code among their colleagues, 14 noted that awareness was extremely limited. Nine of these respondents indicated that awareness existed only among specialized colleagues focusing on health human resources or migration and five reported awareness of the Code's overall purpose but not its contents. This was noted by a civil society respondent from the UK, who said that “few of my colleagues either in the National Health Service or academia are aware of the Code at all, far less having any understanding of its purpose and content” (UK.CS.01). Another civil society respondent from the USA agreed, saying “Only those few colleagues who work specifically on global human resources for health issues are aware of the Code. Among health policy and health services research colleagues there is little or no awareness” (US.CS.09).

By country, UK respondents reported the most awareness (83%; n = 5), while American respondents reported the least (25%; n = 4). Of the four respondents who reported being aware of the Code’s purpose and contents, all were from Australia or the UK and three worked for private companies. By sector, 47% of civil society respondents (n = 14) and 38% of private sector respondents (n = 3) reported awareness of the Code. No government sector respondents reported awareness of the Code among their colleagues. Lacking promotional efforts for the Code are noted by an American government respondent:

“I have heard no reference to the WHO Global Code here at [my organization], which is a Federal agency within the U.S. Department of Health and Human Services. There may have been discussions in other areas of the agency that deal with non-physician workforce issues such as the nursing or public health workforce, but agency-wide there has been no communication sent out, to my knowledge, alerting us to the WHO Code” (US.GS.01).

### Changes resulting from the code

Eighty-six percent of respondents reported that the Code has not had any meaningful impact on their country’s health workforce recruitment practices, policies or regulations (n = 36). Only 7% of respondents reported specific changes in their field of work that were catalyzed by the Code (n = 3), although 19% said it may be too early to tell whether changes have occurred (n = 8). One Canadian civil society respondent identified changes at the provincial level, and a British private sector respondent reported that their organization had been requested to support the government in implementing the Code. No changes were reported from any government respondents.

Some respondents reported that policy changes like those the WHO Code was hoping to inspire had already been made in response to previously-adopted national or regional codes (21%; n = 9). For example, the Commonwealth and Pacific codes were reportedly influential in Australia (n = 6). As an Australian civil society informant noted:

“The WHO Code statement was more reactive than visionary. It merely only encapsulates discussions that had been going on for at least a decade, and a number of 'actions' were probably already in train…My colleagues concerned with the study of movement of health personnel within the Pacific region appear to be aware of the WHO Code which they regard as a follow-on from the Commonwealth and Pacific codes” (AU.CS.03).

The UK Department of Health’s Code of Practice was similarly reported to have triggered earlier immigration and recruitment changes in the UK, as explained by a British civil society respondent:

“The UK has previously implemented a Code of Conduct for ethical recruitment and also changes to postgraduate medical education in which [European Economic Area] (EEA) graduates were prioritised over non-EEA graduates. These have probably had far more impact than the WHO Code” (UK.CS.05).

Key informants also noted implementing policy changes in response to profession-specific codes. Those working in nursing made reference to the International Council of Nurses’ Position Statement on Ethical Nurse Recruitment (2001) and the Canadian Nurses Association’s Position Statement on Ethical Nurse Recruitment (2007) when recalling changes, while those working with physicians referred to the World Organization of Family Doctors’ Melbourne Manifesto (2002).

Forty percent of respondents reported anticipating future changes to their work as a result of the Code (n = 17). Anticipated changes include the development of regulatory policies/legislation (n = 2), addressing the domestic maldistribution of health workers (n = 2), increased data collection (n = 2), and advocacy efforts related to health workforce recruitment (n = 4). At the national level, notable anticipated changes include the development of a national recruitment strategy in Australia (n = 1), stakeholder meeting in the UK (n = 2), and the formation of a working committee on international health workforce recruitment in the USA (n = 1). But the complex nature of health workforce recruitment was reported to have impeded changes. As one respondent from the American private sector noted:

“We proposed an activity related to the Code, but we were asked to remove it by our USAID [United States Agency for International Development] managers. USAID does not have the mandate to work on domestic issues. The problem is that this issue is both foreign and domestic” (US.PS.05).

When specifically asked about changes to recruitment policies, five respondents reported that the Code changed how health workers are recruited to their country (12%). Four of those five respondents were from the UK’s private sector and noted their government’s plans to increase monitoring (n = 2) and produce an annual report on migration trends (n = 2).

### Suggestions to improve the code’s impact

Every respondent offered suggestions to improve the impact of the Code on national and sub-national decision-making (n = 42). For example, when considering future amendments to the Code, respondents cited the importance of using stronger language (n = 3), incorporating stricter enforcement mechanisms (n = 5), citing more supporting research evidence (n = 2), and highlighting best practice exemplars (n = 4) as means to enhance the Code’s impact (n = 14). The need for increasing specificity of the Code’s terms was explained by one American civil society respondent:

“It would be useful to have a clear objective in the WHO Code that relates to specific aspects of international recruitment. As it stands, the language is very broad and refers to strengthening [human resources for health]. There needs to be clarification of what behaviours by whom should change” (US.CS.09).

Thirty-eight percent of respondents believed that complementary guidelines would be helpful in informing the Code's national implementation (n = 16), especially if they were context-specific (n = 5). Technical guidance from international actors like WHO was also suggested, especially for those countries lacking institutional support for the Code’s implementation. As one American government respondent explained: “There is currently no national U.S. regulatory body charged with developing country-wide health workforce policies” (US.GS.01).

### Effectiveness of non-binding codes in general

Eighty-three percent of respondents reported their belief that non-binding codes had limited (31%; n = 13) or no effect (52%; n = 22) on decisions in their country (n = 35). Respondents identified the prioritization of market considerations (n = 4), non-binding nature of these codes (n = 4), and a limited sense of urgency (n = 5) as the most common reasons for their restricted ability to influence decisions. As a Canadian civil society respondent said:

“[The Code] might help, but would need to take into consideration the multiple contexts from which people’s recruitment efforts extend. For example, a hiring committee in small town Canada is highly unlikely to have knowledge of such a Code, and if they did, they probably would not know what to do with it, given their urgent needs for say, a family physician” (CA.CS.01).

Yet 60% of all respondents (including 70% of civil society respondents) also reported that non-binding codes can in theory have some effect (n = 25), either by serving as a basis for policymaking (n = 4), a source of moral imperatives to act (n = 3), or an advocacy tool for political prioritization (n = 4).

## Discussion

### Principal findings and policy implications

Despite persistent calls to regulate the international recruitment of health workers, the vast majority of respondents in this study reported no meaningful impact of the WHO Global Code of Practice within 8–10 months of its adoption on international health workforce recruitment policies, practices, or regulations within their countries. Furthermore, most individuals reported no awareness of the Code within their organizations and that awareness of the Code existed only among their most specialized colleagues. This finding suggests that there may be a gap between demands for action by stakeholders at global forums and the awareness and behaviour of national and sub-national actors. It also suggests that time, publicity and support activities are needed to reach all important audiences, and that the mere adoption of international non-binding codes is not by itself sufficient to induce changes at the national or sub-national level. Low degrees of awareness and information exchange could also be attributed to the lack of transnational advocacy groups that stand to benefit from the Code in developed countries [51].

In cases where country-, region- or profession-specific declarations on health worker recruitment were adopted prior to the Code, respondents attributed policy changes to previous declarations and believed the WHO Code to have no additional effect. This suggests that earlier codes, even if non-binding or adopted by a smaller group, can actually influence national and sub-national decision-making. It also suggests that earlier codes may have more influence than later instruments that were adopted after achieving a global consensus, or that global codes may have less impact when they are less timely or when country- or region-level instruments already exist. Alternatively, this finding suggests that more specific codes – whether targeting a particular country, region or profession – may have greater impact than global all-encompassing instruments which are currently in vogue, perhaps pointing to the perceived importance and influence of regional allegiance and professional authority in changing national and sub-national actors’ behaviour. This may also be relevant for informing efforts to implement the WHO Code, for which country-specific technical guidelines, as well as regional efforts and professional association advocacy, may be particularly helpful.

### Strengths and limitations of the study

This study has five main strengths. First, respondents were purposively drawn from across government, civil society and private sectors in the four English-speaking developed countries with the greatest numbers of migrant physicians and nurses. Second, respondents were mostly very senior-ranking officials who were knowledgeable about health workforce recruitment and their respective sectors. Third, rich qualitative data was collected and analyzed to achieve a deep understanding of the Code’s impact among national and sub-national actors operating in different areas. Indeed, eight out of nine questions in the survey were open-ended, allowing participants to provide more precise and complete information. Fourth, specialists in health workforce migration were consulted throughout this study to ensure that the design and interpretation benefited from their content expertise. Fifth, this study was specifically designed to assess the WHO Code while also gathering insights that may inform efforts to adopt new non-binding codes addressing various challenges in the future.

This study has two main limitations. First, the survey could not use a probability sample and received replies from only 42 of 334 potential respondents, introducing an unknowable amount of sampling error and participation bias. However, this concern is mitigated by the international and sectoral diversity of the sampling frame and respondents, the relatively senior positions they held, and the commonality of their responses (particularly among government and civil society respondents) such that different or additional key informants may not have answered the survey questions any differently. In addition, if anything, the lower response rate may have artificially inflated the (relatively low) percentage of respondents with knowledge of the Code because those potential participants who chose not to respond are presumably less likely to be aware of its existence. Second, the key informants were surveyed only 8–10 months after the Code was adopted in May 2010 and prior to the release of WHO’s draft guidelines for monitoring the Code’s implementation in March 2011 [52]. While this timing may not have allowed sufficient time to observe any impacts, the findings are indicative of national and sub-national decision-makers’ initial perceptions of the Code, its short-term influence, and its potential long-term impact given that the greatest discussion of new instruments presumably occurs in the months immediately following their adoption.

## Conclusions

This study represents the first empirical impact evaluation of the Code’s impact on the behaviour of national and sub-national actors in an effort to inform implementation efforts and provide a baseline for comparisons over time. Despite pressing demands for globally regulating the international recruitment of health workers, there is currently only limited awareness of the Code among national and sub-national actors involved in recruitment to the four English-speaking developed countries with the greatest numbers of migrant health workers. Awareness for and prioritization of particular health issues at the global level does not guarantee awareness at the national or sub-national level.

It is clear that continued efforts are necessary to raise awareness for the Code and support its implementation, including country-, region- and occupation-specific initiatives and utilization of the Code in other instruments and initiatives. As the institutional force behind its adoption, WHO may be well-positioned to provide leadership and technical guidance for this area to the full range of relevant stakeholders. It has already demonstrated its desire and capacity to lead in this area by coordinating the First, Second and Third Global Forums on Human Resources for Health (2008/2011/2013), [48,53] developing evidence-informed policy recommendations for increasing access to health workers in remote and rural areas through improved retention (2010), [14] and hosting a technical briefing on the Code at the 64^th^ World Health Assembly (2011) [54]. Although it is uncertain whether WHO’s continued leadership will be possible in light of recent budget and staffing cuts at the organization [55,56].

But regardless of whether and how the Code’s implementation is supported, additional research is necessary to lend insight into the broader factors that determine the influence of non-binding instruments like the WHO Code, the circumstances under which they are most effective, and the way in which they can be drafted for maximum impact. For example, given that many respondents indicated that national policies changed in response to previously adopted national and regional codes of practice, further studies would be helpful on whether it is the timeliness, geographic relevance, occupation-specificity, or another attribute of these previous codes that encouraged their uptake by decision-makers. Also important is the measurement of time that it takes for global norms and research evidence, as encapsulated in non-binding global instruments like the WHO Code, to be translated into national and sub-national policy and practice.

## Endnote

^a^Key informants were asked to rate their level of agreement with the following statement: “The WHO’s Global Code of Practice on the International Recruitment of Health Personnel has had a meaningful impact on health workforce recruitment, practices, policies, or regulations in my country.” See Web Additional file 1 to view the full questionnaire.

## Competing interests

Steven Hoffman was employed by the World Health Organization’s Department of Human Resources for Health when the WHO Global Code of Practice on the International Recruitment of Health Personnel was being developed. Jennifer Edge was an intern with the World Health Organization’s Department of Human Resources for Health in their Health Workforce Migration and Retention programme when guidelines for monitoring the implementation of the WHO Global Code of Practice on the International Recruitment of Health Personnel were under review. Staff at WHO were asked to provide feedback on the study design and manuscript, but they neither had a veto nor any decisive influence on any part of the study, including the decision to publish and the preparation of the manuscript, which all rested solely with the authors. The opinions expressed in this paper are those of the authors writing in their academic capacities and do not necessarily represent the views of their affiliated institutions.

## Authors’ contributions

JSE contributed to the study design, wrote the first draft of the manuscript, developed the study questionnaire, conducted key informant interviews, performed the data analysis and edited the manuscript. SJH contributed to the study design, developed the survey questionnaire, provided detailed feedback on working drafts of the manuscript and edited the final version of the manuscript. Both authors read and approved the final manuscript.

## Authors’ information

Thank you to Pascal Zurn, Carmen Dolea and Jean-Marc Braichet for providing feedback on the study design and/or an earlier draft of this manuscript.

## Supplementary Material

Additional file 1 Survey questionnaire. Click here for file
