# Supplementary material for: Empirical impact evaluation of the WHO Global Code of Practice on the International Recruitment of Health Personnel in Australia, Canada, UK and USA
Source: Global Health. 2013 Nov 14;9:60. doi: 10.1186/1744-8603-9-60 (PMC3901788; doi:10.1186/1744-8603-9-60)
Supplement: Additional file 1 — Survey questionnaire. [file 1744-8603-9-60-S1.doc]

**Additional file 1: Survey Questionnaire**

1. From your perspective, to what extent are your colleagues generally aware of the WHO Global Code of Practice on the International Recruitment of Health Personnel in your field of work (consider knowledge of the Code’s purpose and contents)?

2. Are you aware of any anticipated changes to take place within your country as a result of the WHO Code of Practice (e.g. influence decisions relating to health policy, health professional regulation, health facility administration, recruitment practices)?

3. Do you know of any particular examples where the WHO Code of Practice influenced specific changes? Please describe any examples that come to mind.

4. From your perspective, has the WHO Code of Practice resulted in any changes to the way that health workers are recruited to your country? If so, how are health workers recruited differently? If not, do you know why changes did not take place (e.g. too soon to implement recommendations, processes were already compliant with the Code, changes were made previously in response to other factors, recommendations in the Code were not feasible)?

5. From your perspective, in what ways has your own work changed as a result of the WHO Code of Practice or as a result of policy changes that were made directly due to the WHO Code of Practice? If no changes have occurred so far, do you expect any changes in your personal work will take place in the future?

6. Based on your personal knowledge and experience, please rate your level of agreement with the following statement by marking a “X” beside the numerical value associated with your rating [please mark only one “X”]: “The WHO’s Global Code of Practice on the International Recruitment of Health Personnel has had a meaningful impact on health workforce recruitment, practices, policies, or regulations in my country.”

_____Strongly Disagree [1]

_____Moderately Disagree [2]

_____Slightly Disagree [3]

_____Neither Agree nor Disagree [4]

_____Slightly Agree [5]

_____Moderately Agree [6]

_____Strongly Agree [7]

7. From your perspective, are there any general or specific changes that could be made to the WHO Code of Practice to improve its impact on the health workforce recruitment, practices, policies, or regulations in your country? Please describe any amendments that come to mind.

8. From your perspective, do you think the Code of Practice would have more impact if complementary guidelines were available to inform implementation efforts?

9. From your perspective, do you think these kinds of voluntary, non-binding global codes of practice are effective instruments for influencing change in your country? Please explain why or why not.
